# Editorial: Oral mucosal immunity: homeostasis and inflammation

**DOI:** 10.3389/fimmu.2023.1214926

**Published:** 2023-06-06

**Authors:** Dunfang Zhang, Junji Xu, Zhi Wang, Hiroko Nakatsukasa

**Affiliations:** ^1^ Department of Biotherapy, State Key Laboratory of Biotherapy and Cancer Center, Collaborative Innovation Center of Biotherapy, West China Hospital, Sichuan University, Chengdu, Sichuan, China; ^2^ Laboratory of Tissue Regeneration and Immunology and Department of Periodontics, Beijing Key Laboratory of Tooth Regeneration and Function Reconstruction, School of Stomatology, Capital Medical University, Beijing, China; ^3^ Immunology Research Center for Oral and Systemic Health, Beijing Friendship Hospital, Capital Medical University, Beijing, China; ^4^ Hospital of Stomatology, Guanghua School of Stomatology, Guangdong Provincial Key Laboratory of Stomatology, and Zhongshan School of Medicine, Sun Yat-Sen University, Guangzhou, Guangdong, China; ^5^ Laboratory of Microbiology and Immunology, Graduate School of Pharmaceutical Sciences, Chiba University, Chiba, Japan

**Keywords:** oral immunity, immune homeostasis, oral mucosa, oral inflammatory disorder, oral inflammation

As the opening of the digestive tract, the on-going mechanical damage caused by chewing leads to continual inflammatory challenges and sustained immune responses to oral microorganisms and food (1). Oral mucosal inflammatory diseases, including infectious diseases (e.g. gingivitis, oral candidiasis, and oral herpes) and non-infectious inflammatory diseases (e.g. oral lichen planus (OLP) and recurrent aphthous ulcer (RAU)) are common and frequently occurring diseases of oral mucosa and may seriously affect our oral health. Besides, many systemic inflammatory diseases have typical oral inflammation phenotypes. During the past decade, a number of key progresses have been made in the study of oral mucosal immune homeostasis and diseases (2). By now, it has been well proven that oral mucosal immunity could affect the immune responses in the gastric intestinal tract and throughout the whole body (3). Thus, the goal of this Research Topic is to collect the latest research advances regarding the regulation of oral mucosal immunity in health and disease. To this end, we hosted 13 original research articles, review articles, and mini reviews.

Oral ulcer is the most common inflammatory disease in the oral cavity. To investigate whether N6-methyladenosine (m6A)-related single nucleotide polymorphisms (m6A-SNPs) are involved in the pathogenesis of oral ulcers, Wu et al. analyzed genome-wide association studies (GWAS) database and identified 11 m6A-SNPs that were related to oral ulcers, showing m6A RNA transcription modification may be involved in the development of oral ulcers.

OLP is a chronic inflammatory disorder of the oral mucosa (4). The clinical symptoms of OLP and discoid lupus erythematosus (DLE) are very similar. Wang R. et al. summarized the significant differences in the expression levels and genotype polymorphism of two cytokines, TNF-α and IL-10, in OLP and DLE. They concluded that differential genotypes of TNF-α and IL-10 could be an immunological diagnosis for these two diseases.

Currently, the treatment of OLP mainly relies on immunosuppressive drugs (5). It has been proven that the response of OLP patients to the common immunosuppressive therapy is heterogeneous (6). To predicate the effectiveness of immunosuppressive therapy in OLP, Xu et al. developed a workflow by acquiring image-based features in OLP. They found that the best performance prediction model built by logistic regression showed an accuracy of 90%. This model could provide a valuable reference for the choice of medication for the OLP clinical treatment. Besides traditional immunosuppressive drugs, novel immunotherapy approaches are gaining traction. Xue et al. found that regulatory T cell (Treg cell) therapy may be an effective new treatment strategy for oral inflammatory diseases such as OLP.

The oral cavity has a rich symbiotic microbiome, second only to that of the gut (3). Long et al. revealed that the microbiota might maintain oral homeostasis by reshaping the structure of the oral epithelial barrier and changing the function of molecular biology. Wang X. et al. investigated the association between gut microbiota alterations and recurrent aphthous ulcer (RAU), and they indicated that gut dysbacteriosis, microbial dysfunction and immune imbalance occurred in RAU patients.

The incidences of oral mucosal inflammatory diseases are associated with a variety of systemic diseases (7). Zhang et al. found that the prevalence of Hashimoto’s thyroiditis (HT) in OLP patients, especially in female OLP patients, is significantly higher than that in the general population. Li et al. reviewed the findings between oral mucosal inflammation and ulcerative colitis (UC), and they concluded that pyostomatitis vegetans, RAU and periodontitis, could not only be used to be risk factors for disease occurrence of UC, but also could be used to predict disease severity of UC. More than that, Seidel et al. investigated levels of inflammatory factors in newborns with orofacial clefts (OFC), and found that the expression of several inflammatory factors was increased than that of the healthy controls, suggesting that these children were at risk for oral mucosal inflammation.

So far, the pathogenesis of various oral mucosal inflammatory diseases is still unclear. Epithelial-mesenchymal transition (EMT) is a crucial biological process in the pathogenesis of oral mucosal disorders. Meng et al. provided a comprehensive evaluation of type-2 EMT in chronically inflammatory oral mucosal disorders, and they believe that targeting EMT could be a promising novel strategy to treat oral mucosal disorders in the future. Besides EMT, cellular senescence caused tissue aging is also thought to be a key factor in oral inflammation. Villalobos et al. summarized the effects of aging on periodontal tissues, and concluded that it could cause the imbalance of the periodontium and periodontitis. Yue et al. summarized the studies of the senescence-associated secretory phenotype (SASP) in in oral immunity, and they found that SASP might play a pleiotropic role in the pathogenesis of oral immunity.

In addition to oral mucosa, the immune homeostasis of oral secretory glands such as salivary glands is also very important for health. Sjogren’s syndrome (SS) is a chronic autoimmune disorder that seriously affects the quality of life of patients (8). Zhan et al. reviewed the pathogenesis and treatment of SS, and they emphasized that targeted drugs, low-side-effect drugs, and combination therapies should be the focus of future research.

In summary, this Research Topic collected the current advances regarding the oral mucosal immune regulation, oral mucosal inflammatory disease pathogenesis and novel therapy strategies. More and more evidence shows that oral mucosal immune homeostasis is not only indispensable for health of the oral cavity, but also important for systemic health. Future studies should focus on two aspects: one is to explore the relationship between oral mucosal immune homeostasis and systemic immune homeostasis; the other is to study how to apply the findings of oral mucosal immunopathogenesis and novel therapy strategies to clinical treatment.

## Author contributions

DZ and HN wrote the manuscript. All authors reviewed and edited the manuscript, and approved it for publication.

## References

[1] Dutzan N Abusleme L Bridgeman H Greenwell-Wild T Zangerle-Murray T Fife ME. On-going mechanical damage from mastication drives homeostatic Th17 cell responses at the oral barrier. Immunity (2017) 46(1):133–47. doi: 10.1016/j.immuni.2016.12.010 PMC5263257 28087239

[2] Williams DW Hajishengallis G Moutsopoulos NM. Regional specification of oral mucosal immunity. Sci Immunol (2022) 7(72):eabp8632. doi: 10.1126/sciimmunol.abp8632 35714199 PMC10498435

[3] Kitamoto S Nagao-Kitamoto H Hein R Schmidt TM Kamada N. The bacterial connection between the oral cavity and the gut diseases. J Dent Res (2020) 99(9):1021–9. doi: 10.1177/0022034520924633 PMC7375741 32464078

[4] Wang H Zhang D Han Q Zhao X Zeng X Xu Y. Role of distinct CD4(+) T helper subset in pathogenesis of oral lichen planus. J Oral Pathol Med (2016) 45(6):385–93. doi: 10.1111/jop.12405 26693958

[5] Zhang D Wang J Li Z Zhou M Chen Q Zeng X. The activation of NF-kappaB in infiltrated mononuclear cells negatively correlates with treg cell frequency in oral lichen planus. Inflammation (2015) 38(4):1683–9. doi: 10.1007/s10753-015-0145-x 25761427

[6] Chamani G Rad M Zarei MR Lotfi S Sadeghi M Ahmadi Z. Efficacy of tacrolimus and clobetasol in the treatment of oral lichen planus: a systematic review and meta-analysis. Int J Dermatol (2015) 54(9):996–1004. doi: 10.1111/ijd.12925 26204904

[7] Kleinstein SE Nelson KE Freire M. Inflammatory networks linking oral microbiome with systemic health and disease. J Dent Res (2020) 99(10):1131–9. doi: 10.1177/0022034520926126 PMC7443998 32459164

[8] Xu J Wang D Liu D Fan Z Zhang H Liu O. Allogeneic mesenchymal stem cell treatment alleviates experimental and clinical sjogren syndrome. Blood (2012) 120(15):3142–51. doi: 10.1182/blood-2011-11-391144 PMC3471521 22927248

